# The Prevalence of Pharmacological Neuroenhancement Among University Students Before and During the COVID-19-Pandemic: Results of Three Consecutive Cross-Sectional Survey Studies in Germany

**DOI:** 10.3389/fpubh.2022.813328

**Published:** 2022-03-24

**Authors:** Pavel Dietz, Antonia M. Werner, Jennifer L. Reichel, Markus Schäfer, Lina M. Mülder, Manfred Beutel, Perikles Simon, Stephan Letzel, Sebastian Heller

**Affiliations:** ^1^Institute of Occupational, Social and Environmental Medicine, University Medical Centre of the Johannes Gutenberg University, Mainz, Germany; ^2^Department of Psychosomatic Medicine and Psychotherapy, University Medical Center of the Johannes Gutenberg University, Mainz, Germany; ^3^Department of Communication, Johannes Gutenberg University, Mainz, Germany; ^4^Department of Work, Organizational and Business Psychology, Institute of Psychology, Johannes Gutenberg University, Mainz, Germany; ^5^Department of Sports Medicine, Rehabilitation and Disease Prevention, Institute of Sport Science, Johannes Gutenberg University, Mainz, Germany

**Keywords:** university, college, epidemiology, brain doping, neuroenhancement (NE)

## Abstract

**Background:**

According to the literature, the conditions of studying and living as well as the psychological, social and health behavior-related variables, which were strongly related to pharmacological neuroenhancement (PN) before the pandemic, significantly changed during the pandemic. For this reason, it is expected that the prevalence of PN among university students is higher during the pandemic compared to before the pandemic. Therefore, the present study aimed to investigate and compare the prevalence of PN among university students before and during the COVID-19-pandemic.

**Methods:**

Three online surveys assessing the 12-month prevalence of PN were conducted among university students at the University of Mainz, Germany. The first survey took place in summer term 2019 (before the pandemic), the second in summer term 2020 (during the first German lockdown), and the third in summer term 2021 (after the second German lockdown). Pearson's chi-square test was used to test whether the 12-month prevalence of PN differed significantly between the three surveys.

**Results:**

The 12-month prevalence of PN was 10.4% in 2019, 11.3% in 2020, and 8.0% in 2021. Chi-square tests revealed no statistical difference in the prevalence of PN between 2019 and 2020. Overall, the use of PN was lower in 2021 compared to 2019 (*p* < 0.0001) as well as in comparison to 2020 (*p* = 0.001). Only the use of cannabis slightly increased from 2019 to 2020 (7.1 vs. 8.3%) and decreased in 2021 (5.4%). At all three time points, cannabis was the most commonly used substance for the purpose of PN. Consequently, the results suggest that the prevalence of PN was highly intertwined with the prevalence of cannabis use for PN.

**Discussion:**

The decrease in the prevalence of PN of around three percentage points in 2021 compared to the previous years was a surprising finding. It may be mainly due to the decrease in the prevalence of cannabis for the purpose of PN. However, the fairly high prevalence of PN of around 8% in 2021 is still an important finding that demonstrates that there is still an urgent need for prevention initiatives among university students to combat the use of PN.

## Introduction

Pharmacological neuroenhancement (PN) is generally defined as the use of illicit or prescription drugs by healthy individuals for cognitive-enhancing purposes such as enhancing alertness, attention, concentration, memory, and mood (1, 2). Daubner et al. (3) give a more in-depth look at the development and discussion regarding the different, partly popular scientific terminology and paraphrases around PN. In western Europe and the United States, epidemiological studies showed that PN is prevalent specific occupational settings such as surgeons and economics (4–6) and in the general population (7–9). Furthermore, a considerable number of studies demonstrated the use of PN in the collective of university students. For example, as lifetime prevalence for PN, 7.8, 3.2, and 19.2% was reported among Swiss (10), Norwegian (11), and British (12) students. Using an indirect survey technique, Dietz et al. (13, 14) described estimates for the 12-month prevalence of PN between 12 and 20% among university students from Austria and Germany. These estimates varied between the different study disciplines. Moreover, within a comprehensive review and meta-analysis, Benson et al. (15) reported the 12-month prevalence for the use of prescription stimulants to lay between 5 and 35% among college students in the US, demonstrating considerable heterogeneity in the range of this prevalence.

From a public health point of view, the use of PN is seen critically because it appears to be associated with physiological and psychological side effects and increased mortality, can lead to addiction, and may provide a gateway for the use of other substances (16–21). Therefore, the need for prevention of PN has been underlined by several experts (3). In this context, university students were pointed out as a population of specific relevance, since university students are tomorrow's leaders, decision-makers, and parents. Consequently, health promotion and prevention in this collective would be sustainable and beneficial for the general society (22, 23). Aiming to develop and implement prevention strategies of PN among university students more specifically, Heller et al. (24) investigated potential sociodemographic and study-related risk groups as well as predictors of PN taking sociodemographic, psychological, study-related, general psychosocial factors, as well as health behavior-related variables into account. They concluded that specific health behavior variables such as physical activity or nutrition had the most decisive influence on the explained variance of PN, supporting the results of previous studies (13, 25). In addition, other studies identified psychological factors such as stress (26–28) and study-related psychosocial factors such as perceived academic benefits (29–32) being related to PN.

On January 7th, 2020, Chinese authorities identified a novel coronavirus (SARS-CoV-2). Due to the rapid increase in cases of the corresponding coronavirus disease 2019 (COVID-19) worldwide, the World Health Organization officially declared the spread of COVID-19 as a pandemic (33). In response to the pandemic, universities in Germany were closed in March 2020 aiming to positively influence the course of infection. The abrupt loss of personal contacts with peers and faculty, postponement of curricula, research, practical work, and exchange programs, profound changes regarding their financial and housing situation as well as the abrupt switch to online learning (34–36) happened with far-reaching consequences, not only for the education of students but also for their mental health, health behavior and social behavior. For example, using a longitudinal design, Werner et al. (37) showed that university students' levels of loneliness and depression, symptoms of anxiety, and somatic complaints increased during the pandemic, supporting the results of previous studies from the USA and China (38, 39). With regard to behavioral variables, Csépe et al. (40) concluded that social behavior (e.g., fear and adherence to rules) and health-related behavior (e.g., smoking, nutrition, and physical activity) of university students changed in a negative way during the pandemic (40).

In summary, many empirical studies showed that PN was prevalent among university students before the COVID-19-pandemic. Furthermore, a wide range of explanatory variables of PN were examined before the pandemic, ranging from psychological, social, study-related, and health behavioral variables. However, with regard to the prevalence of PN among university students during the pandemic, we are not aware of any internationally published article addressing this issue. Since the conditions of studying and living as well as the psychological, social, and health behavior-related variables, which were strongly related to PN before the pandemic, significantly changed during the pandemic, it is expected that the prevalence of PN among university students is higher during the pandemic compared to before the pandemic. Therefore, the present study aimed to address this knowledge gap by investigating and comparing the prevalence of PN among university students before and during the COVID-19-pandemic.

## Methods

Three online surveys were conducted among university students at the University of Mainz, Germany, as part of the interdisciplinary research project Healthy Campus Mainz. The first survey took place in summer term 2019 between June and August (before the pandemic), the second in summer term 2020 in June (during the first German lockdown), and the third in summer term 2021 between June and August (after the second German lockdown). All three surveys followed the same procedure. Students were invited to participate *via* e-mail addressed to all registered students using the central student mailing list of the university. The questionnaire of the first (pre-pandemic) survey covered questions regarding sociodemographic data, health status, health behavior, and a wide range of potential determinants of health status and health behavior. More specific information concerning the survey procedure and the content of the first survey can be found elsewhere (41). The second and third (pandemic) survey contained additional specific questions with regard to the COVID-19-pandemic. Participation was voluntary and informed consent was obtained before participation. Study approval was obtained by the ethical committee of the Medical Association of Rhineland-Palatinate (No. 2019-14336) for the first study and the Institute of Psychology of the JGU for the second (No. 2020-JGU-psychEK-S008) and the third (2021-JGU-psychEK-S017) study.

PN was investigated in all surveys as part of the health behavior questions following the same methodical approach published for example, by Heller et al. (24) and others (4, 5). Accordingly, the translated question to assess the prevalence PN was: “Have you ever used the following substance/-s without medical necessity, for the purpose of enhancing your cognitive performance or to better handle your studies (not for reasons of enjoyment)?” The following illicit or prescription drugs could be selected via multiple-choice, and each with the scale “never,” “within the last 30 days,” “within the last 12 months,” or “more than 12 months ago”: methylphenidate (e.g., Ritalin^®^), amphetamine preparation (e.g., Adderall^®^), atomoxetine (e.g., Strattera^®^), modafinil (e.g., Provigil^®^), ecstasy, ephedrine, cocaine, illicit amphetamines (e.g., Speed), crystal meth, cannabis, and “other substances.” To be able to investigate potential changes in the prevalence of PN over time, the 12-month prevalence (dichotomous: “yes”/“no”) instead of the lifetime prevalence was used for all further analyses. Pearson's chi-square test was used to test whether the 12-month prevalence of PN differed significantly between the three surveys. The prevalence of PN is presented as proportion of “yes” in the analyzed sample. Descriptive variables of the three surveys are presented as means with standard deviations (SD) for continuous variables and as absolute and relative frequencies numbers and percentages for categorical variables.

## Results

After data cleaning, a total of *N* = 4,351 students participated in the 2019 survey, *N* = 3,066 students in the 2020 survey and *N* = 1,438 students in the 2021 survey. The samples of the three surveys were largely comparable with regard to gender, age, and study-related characteristics (Table 1). The specific question with regard to the prevalence of PN was answered by *N* = 3,984 students in 2019, *N* = 2,796 students in 2020, and *N* = 1,232 students in 2021. The 12-month prevalence of PN was 10.4% in 2019, 11.3% in 2020, and 8.0% in 2021 (Table 2). Chi-square tests revealed no statistical difference in the prevalence of PN between 2019 and 2020. Overall, the use of PN was lower in 2021 compared to 2019 (*p* < 0.0001) as well as in comparison to 2020 (*p* = 0.001). Taking a closer look at the specific substances used for the purpose of PN (Table 3), it can be seen that the 12-month-prevalence rates of all substances were relatively constant at the three time points. Only the use of cannabis slightly increased from 2019 to 2020 (7.1 vs. 8.3%) and decreased in 2021 (5.4%).

**Table 1:** Sample characteristics of the three surveys.

	**2019 (pre-pandemic, *N* = 4,351)**	**2020 (during pandemic, *N* = 3,066)**	**2021 (during pandemic, *N* = 1,438)**
Gender, *n* (%)	(*n* = 4,350)	(*n* = 3,066)	(*n* = 1,436)
Female	3,065 (70.4)	2,225 (72.6)	1,065 (74.2)
Male	1,246 (28.6)	821 (26.8)	338 (23.5)
Diverse	39 (0.9)	20 (0.7)	23 (2.3)
Age, years (mean ± SD)	16–73 (23.8 ± 4.4)	16–68 (23.4 ± 4.4)	16–69 (23.7 ± 4.7)
Semester (mean ± SD)	1–45 (7.1 ± 4.9)	1–35 (6.4 ± 4.5)	1–38 (6.5 ± 4.7)
Aspired degree, *n* (%)	(*n* = 4,351)	(*n* = 3,065)	(*n* = 1,436)
Bachelor	2,261 (52.0)	1,709 (55.8)	827 (57.6)
Master	920 (21.1)	645 (21.0)	269 (18.7)
State examination	977 (22.5)	662 (21.6)	317 (22.1)
Other	193 (4.4)	49 (1.6)	23 (1.6)
Field of study, *n* (%)	(*n* = 4,342)	(*n* = 3,012)	(*n* = 1,430)
STEM^a^	783 (18.0)	506 (16.8)	217 (15.2)
Social sciences, media or sport	774 (17.8)	493 (16.4)	269 (18.8)
Linguistics, humanities, and cultural sciences	871 (20.1)	621 (20.6)	315 (22.0)
Medicine	582 (13.4)	341 (11.3)	211 (14.8)
Law and economics	576 (13.3)	479 (15.9)	156 (10.9)
Teaching	665 (15.3)	510 (16.9)	243 (16.9)
Other	91 (2.1)	62 (2.1)	19 (1.3)

a *Science, Technology, Engineering, and Mathematics*.

**Table 2:** Twelve-month prevalence of PN in the three surveys.

	**2019 (pre-pandemic)**	**2020 (during pandemic)**	**2021 (during pandemic)**
All participants, *n*
	3,984	2,796	1,232
12-month prevalence, *n (%)*	416 (10.4)	316 (11.3)	98 (8.0)

**Table 3:** Prevalences for the use of each specific illicit or prescription drug for PN in the three surveys (*N* = 3,984 in 2019; *N* = 2,796 in 2020; *N* = 1,232 in 2021).

	**12-month prevalence for the use of specific substances for PN**		
	**2019 (pre-pandemic)**	**2020 (during pandemic)**	**2021 (during pandemic)**
**Prescription and illicit drugs**
Methylphenidate	1.4% (*n* = 54)	1.5% (*n* = 46)	1.4% (*n* = 17)
Amphetamine preparation	0.2% (*n* = 7)	0.3% (*n* = 8)	0.2% (*n* = 3)
Atomoxetine	0.2% (*n* = 6)	0.2% (*n* = 5)	0.2% (*n* = 3)
Modafinil	0.3% (*n* = 13)	0.2% (*n* = 6)	0.2% (*n* = 3)
Ecstasy (MDMA)	1.0% (*n* = 38)	0.8% (*n* = 23)	0.6% (*n* = 8)
Ephedrine	0.2% (*n* = 8)	0.1% (*n* = 3)	0.2% (*n* = 3)
Cocaine	0.6% (*n* = 25)	0.9% (*n* = 25)	0.6% (*n* = 7)
Amphetamine	0.9% (*n* = 36)	0.9% (*n* = 25)	0.6% (*n* = 7)
Crystal meth	0.1% (*n* = 3)	0.1% (*n* = 4)	0.0% (*n* = 0)
Cannabis	7.1% (*n* = 284)	8.3% (*n* = 230)	5.4% (*n* = 67)
Other substances	2.2% (*n* = 91)	2.8% (*n* = 77)	1.8% (*n* = 22)

## Discussion

In the present study, we investigated whether the 12-month prevalence of PN among university students was higher during the COVID-19-pandemic compared to the prevalence before the pandemic. Therefore, three waves of survey in the summer terms of the respective years were conducted, one before the pandemic (2019), one during the first German lockdown (2020), and one after the second German lockdown (2021), when the infection case rates were continuously decreasing in Germany and lockdown measures were loosened. The sample sizes of the three surveys decreased from 2019 to 2020 and from 2020 to 2021. As we used the same methodological approach for recruiting university studens in all three surveys by contacting all students of the University of Mainz per E-Mail *via* a central mailing list (41), we do not think that the decrease in sample size had methodological reasons. However, we noticed (although empirical data are lacking for this hypothesis) an increase in “tiredness” of being online most time of the day for example for working, studying, and social interactions. Consequently, we hypothesize that the university students lost their motivation to participate in one more voluntary online survey during the pandemic what may be a reason for the decrease in sample size.

Contrary to our expectation, the prevalence of PN was relatively constant in 2019 and 2020 but decreased significantly in 2021. At all three time points, cannabis was the most commonly used substance for the purpose of PN, which made up around two-thirds to three-fourths of the total prevalence of PN at all measurement points. Consequently, the results suggest that the prevalence of PN was highly intertwined with the prevalence of cannabis use for PN.

The relatively constant or slightly increasing numbers from 2019 and 2020 are in line with the recently published drug survey 2021 of the federal government, indicating that the prevalence of the use of cannabis among young adults is continuously increasing since the last years (42). Furthermore, as stated in the world drug report 2021 of the United Nations, cannabis use patterns had remained relatively stable during the first lockdown period in the European Union, with nearly half of the participants reporting no change in their cannabis use, compared with the pre-lockdown period. In addition, as described in the second booklet on the global overview of drug demand and drug supply of the world drug report 2021, supply chains for Cannabis and also for other psychoactive substances were not affected by the pandemic. In the fifth booklet of this report on the impact of COVID-19 on drugs, it is further stated that COVID-19 may have accelerated the pre-existing trends toward increased use and availability of cannabis in some high-income countries as some people have turned to the drug to alleviate stress or manage boredom brought on by stay-at-home orders (43). However, these reports refer to the prevalence for the use of cannabis *per se* and not for the specific purpose of PN, as we did in our study. Therefore, the comparability of numbers has to be seen with caution.

The decrease in the prevalence of PN of around three percentage points in 2021 compared to the previous years was a surprising finding. It may be mainly due to the decrease in the prevalence of cannabis for the purpose of PN, which was also around three percentage points. In contrast, the prevalence for the other surveyed substances for PN remained the same. However, as literature regarding the prevalence of PN during the COVID-19-pandemic is rare, any discussion of this finding will be mostly hypothetical. One reasoning could be that potential demands (e.g., mental, social, and study-related) that were present during the lockdown and university closure period may have decreased after the end of the lockdown when infection case rates declined and restrictions were continuously loosened. In contrast to this reasoning, a study among college students performed at seven colleges in the United States showed that depressive symptoms and anger were modestly higher post-college closure compared to pre-college closure period, whereas no differences were observed in anxiety symptoms or insomnia and variables of cannabis use. However, the data were subject to both self-report and self-selection bias (44). One theoretical approach to explain the decrease of the prevalence of PN in 2021 could be Kahnemanns's Prospect Theory (45). According to this approach, the slightly higher prevalence of PN at the beginning of the pandemic (2020) could be explained by the situation and circumstances that students were confronted with, which were characterized by many uncertainties such as loss of personal contacts with peers and faculty, research, practical work, and exchange programs, profound changes regarding their financial and housing situation as well as the abrupt switch to online learning. These may have increased tendencies toward risk behaviors like PN. In contrast, the decreased prevalence of PN in 2021 reflects that the experiences after 1 year of studying under the conditions of the pandemic may have given a certain kind of security to the students that studies can be handled and even solutions like online-exams may cause less stress and therefore less risk behviors like PN.

For a more in-depth interpretation of the present results, especially the decrease in the prevalence of PN in 2021 compared to the previous years, more studies are needed addressing the prevalence of PN among university students and potential explanatory variables of PN during the COVID-19-pandemic. Despite the necessity of further research, the fairly high prevalence of PN of around 8% is still an important finding that demonstrates that there is still an urgent need for prevention initiatives among university students to combat the use of PN. To be able to plan evidence-based and effective PN-prevention initiatives for university students, it is important to understand the conditions and factors predicting PN among this target group. In this context, using a stepwise binary logistic regression model, Heller et al. (24) showed that specific varibles of health behavior predicted the use of PN among university students indicating that initiatives strengthening health behavior may prevent PN. This is in line with other research indicating that strengthening health-related key skills and ressources (in the sense of positive coping strategies) leads to a decrease in the prevalence of PN. For example, Bagusat et al. (26) concluded that tailored resilience interventions that improve the ability to adapt to and recover from stressors prevent the use of PN. Consequently, we recommend that initiatives aiming to prevent PN among university students have to be multifactorial taking the specific conditons of studying into account and have to focus on strengthening competences with regard to health behavior, mental health literacy and non-pharmacological ressources and strategies (24, 46, 47). Especially during the COVID-19-pandemic and in times of distance-teaching, online programs are of particular relevance. To name just some concrete examples of evidence-based online intitiatives for university students, at the university of Mainz, Germany, *KEN-Online*, and *STUDYCoach* are programs where students learn to deal with, for example, their emotions, stress, or symptoms of depression or anxiety. Another approach which aims to transport the topics physical activity, sedentary behavior and digital detox into (online) lectures is the program called *Health Express*. Here, long lectures are interrupted by short video clips which address a specific health-related topic and which were specifically developed for the target group university students under participation of university students. Moving from sitting into standing position is obligatory at the beginning of all video clips (48).

As potential limitation, it has to be mentioned that no causal inference can be drawn from cross-sectional data, as performed in the present study.

## Data Availability Statement

The raw data supporting the conclusions of this article will be made available by the authors, without undue reservation.

## Ethics Statement

The studies involving human participants were reviewed and approved by Medical Association of Rhineland-Palatinate (No. 2019-14336) for the first study and the Institute of Psychology of the JGU for the second (No. 2020-JGU-psychEK-S008) and the third (2021-JGU-psychEK-S017) study. Written informed consent from the participants' legal guardian/next of kin was not required to participate in this study in accordance with the national legislation and the institutional requirements.

## Author Contributions

PD, AW, MS, JR, LM, and SH contributed in data collection. PD, AW, and SH contributed in data analysis. PD, PS, and SH contributed in interpretation of the results. PD had the lead in manuscript writing. All authors have read and approved the final draft of the manuscript and contributed in conceptualization and study design.

## Funding

This Healthy Campus Mainz project was funded by BARMER health insurance and carried out with the support of the Johannes Gutenberg-University of Mainz and the University Medical Center of the Johannes Gutenberg-University of Mainz.

## Conflict of Interest

The authors declare that the research was conducted in the absence of any commercial or financial relationships that could be construed as a potential conflict of interest.

## Publisher's Note

All claims expressed in this article are solely those of the authors and do not necessarily represent those of their affiliated organizations, or those of the publisher, the editors and the reviewers. Any product that may be evaluated in this article, or claim that may be made by its manufacturer, is not guaranteed or endorsed by the publisher.
